# Pyruvate Kinase M2 Role in Cardiovascular Repair

**DOI:** 10.3390/cells14201623

**Published:** 2025-10-17

**Authors:** Mohd Rihan, Lior Zangi, Ajit Magadum

**Affiliations:** 1 Center for Regenerative Medicine, USF Health Heart Institute, Department of Internal Medicine, University of South Florida, 560 Channelside Drive, MDD 810B, Tampa, FL 33602, USA; 2 Cardiovascular Research Institute, Icahn School of Medicine at Mount Sinai, New York, NY 10029, USA

**Keywords:** PKM2, cardiac repair, myocardial infarction, cardiac protection, cardiovascular regeneration

## Abstract

Adult cardiomyocytes (CMs) lose their proliferative capacity shortly after birth, posing a major challenge for cardiac repair following injury such as myocardial infarction (MI). Despite significant advances over the past decade, many strategies for promoting cardiac regeneration have faced limitations, underscoring the need to identify novel molecular pathways and targets. Pyruvate kinase muscle isoform 2 (PKM2), a key metabolic enzyme, has emerged as a compelling candidate in this context due to its multifaceted roles in cellular metabolism, proliferation, redox balance, angiogenesis, and master gene regulator in repair. Recent studies highlight the critical function of PKM2 in cardiac repair and regeneration. PKM2 not only promotes the proliferation of CMs but also protects the heart from oxidative stress by redirecting glycolytic intermediates toward the pentose phosphate pathway (PPP), thereby increasing nicotinamide adenine dinucleotide phosphate (NADPH) levels, reducing reactive oxygen species (ROS), and minimizing DNA damage. Moreover, PKM2 interacts with key signaling molecules, including β-catenin, hypoxia-inducible factor 1α (HIF-1a), and checkpoint kinase 1 (CHK1), to promote CM cell cycle reentry, angiogenesis, and enhanced cell survival. Collectively, these multifaceted actions highlight PKM2 as both a metabolic and signaling hub in cardiac repair by promoting myocardial remuscularization, protection, and revascularization and position PKM2 as a promising therapeutic. This review explores the diverse roles of PKM2 in myocardial repair and discusses its potential as a novel avenue for advancing regenerative therapies in cardiovascular medicine.

## 1. Introduction

Cardiovascular diseases (CVDs) include ischemic heart disease, stroke, heart failure, peripheral and aortic arterial disease, arrhythmias, and valvular disorders. CVDs remain the leading cause of mortality worldwide and are a major contributor to global health loss. The prevalence of cardiovascular diseases is projected to increase by 90.0%, with mortality rising by 73.4% and disability-adjusted life years (DALYs) increasing by 54.7% between 2025 and 2050. The number of cardiovascular deaths is estimated to rise from 20.5 million in 2025 to 35.6 million in 2050 [1,2]. However, as per the American Heart Association 2025 heart disease and stroke statistics report, myocardial infarction (MI) remains a major public health concern, with an estimated occurrence every 40 s in the United States [3].

In the normal neonatal heart, glycolysis predominates as the primary source of ATP at birth, accounting for approximately 50% of total cardiac ATP production at postnatal day 1 (P1) and declines to 10% by P7 and 5% in adults [4]. Moreover, a decrease in fatty acid oxidation facilitates a glycolytic metabolic switch, which supports cardiac repair and regeneration [5]. A hallmark metabolic feature of proliferating CMs is their enhanced reliance on glycolysis for ATP production. However, rising oxygen availability promotes a metabolic shift from glycolysis to mitochondrial oxidative phosphorylation during the transition from fetal to postnatal life, which coincides with CM cell cycle withdrawal [6,7,8,9]. In addition to this, modulating metabolic reprogramming between glycolysis and the tricarboxylic acid cycle (TCA) was described by Bae et al., demonstrating that inhibition of the mitochondrial enzyme succinate dehydrogenase (SDH) by malonate extends the window of CM proliferation and cardiac regeneration after birth. Meanwhile, in the adult mouse heart, myocardial infarction results in a robust regenerative response within four weeks, driven by enhanced adult CM proliferation and revascularization [10]. This suggested that metabolic reprogramming toward glycolysis by inhibiting *TCA* promotes CM proliferation, revascularization, and cardiac regeneration.

**Figure 1:**
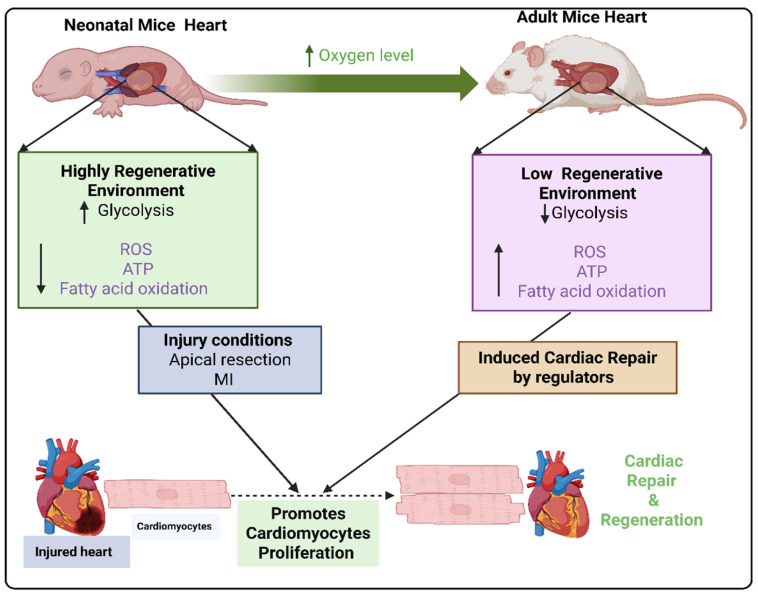
Illustration of the metabolic reprogramming differences in the neonatal and adult mouse hearts. Figure is created with BioRender.com.

Metabolic reprogramming plays a crucial role in cardiac repair and regeneration. Dr. Otto Warburg first described the metabolic reprogramming in cancer cells in the 1920s, which is now known as the Warburg effect [11]. Recently published reports showed a positive correlation between altered metabolic reprogramming and cardiac repair [12]. The modulation of metabolic reprogramming related enzymes can promote CM proliferation and cardiac repair. A report by Chen et al. demonstrated that the mechanism of CM lactate dehydrogenase A (LDHA) mediated metabolic reprogramming. P1 heart showed higher LDHA expression, but its expression decreased during the postnatal period. CM-specific LDHA deletion showed reduced CM proliferation and worse cardiac function. However, CM-specific LDHA overexpression promotes CM proliferation and improved cardiac function via inhibiting succinylation-dependent ubiquitination of thioredoxin reductase 1 (Txnrd1) [13]. In the early stage of post-MI, monocytes undergo metabolic reprogramming by H3K18 histone lactylation on monocytes, leading to dysregulate glycolysis and monocarboxylate transporter-1 (MCT1)-mediated lactate transport [14]. Moreover, metabolic reprogramming by the disablement of fatty acid oxidation in CMs also showed improvement in hypoxia resistance and stimulated CM proliferation, allowing heart regeneration in ischemia–reperfusion injury by activating KDM5 demethylases on H3K4me3 in the MI heart [5]. In addition to that, metabolic reprogramming by altering sphingolipid metabolism has also been found to be involved in cardiac repair. The ceramide level and genes involved in ceramide synthesis (C16, C20, C20:1, and C24) were significantly altered within 24 h after MI. Decreasing the level of ceramide via the gain of function of acid ceramidase (enzyme involved in breaking down of ceramides into sphingosine and fatty acids) through modified RNA (AC-modRNA) treatment showed cardioprotection via increased cell survival, improved cardiac function, smaller scar size, and more prolonged survival after 28 days of post-MI [15]. Moreover, several genes have been reported to promote metabolic reprogramming and cardiac repair. Upregulation of forkhead box 1 (Fox1) Fox2, FGF1/p38 MAP kinase inhibitor, cyclin-dependent kinase 1 (CDK1), CDK4, cyclin B1, D1, D2, and Meis1 extends the postnatal CM proliferative window and promotes cardiac regeneration in adult post-MI heart [16,17,18,19,20]. The genes, such as hypoxia-inducible factor 1α (Hif1α), Hippo pathway, yes-associated protein (YAP), β-catenin, ErbB2 receptor tyrosine kinase 2 (ERBB2), and phosphoserine aminotransferase 1 (PSAT1), have been reported to be involved in metabolic reprogramming and CM proliferation and cardiac repair [21,22,23,24,25,26,27,28]. All this evidence concludes that metabolic reprogramming is essential for cardiac repair, as it shifts energy production from oxidative phosphorylation and fatty acid oxidation toward glycolysis. This metabolic transition supports the energetic and biosynthetic demands of proliferating CM, enhances cell survival under hypoxic conditions, and modulates key signaling pathways involved in tissue regeneration. Among them, PKM2 is one of the emerging promising therapeutic targets, demonstrating significant potential in myocardial repair and regeneration. Figure 1 illustrated the metabolic differences in the neonatal and adult mouse hearts.

## 2. Glycolytic Pyruvate Kinase Muscle Isozyme M2 (PKM2)

The glycolytic pathway is a central metabolic process that exhibits extensive crosstalk with other cellular pathways, including the PPP, tricarboxylic acid (TCA) cycle, serine biosynthesis, and amino acid synthesis [29]. Pyruvate kinase (PK), a key glycolytic enzyme, catalyzes the final step of glycolysis by converting phosphoenolpyruvate (PEP) to pyruvate. There are four isoforms of PK: PKM1, PKM2, PKL, and PKR. In mammals, the genome encodes two distinct pyruvate kinase (PK) genes: PKLR and PKM. The *PKLR* gene gives rise to two isoforms: PKR, expressed in red blood cells, and PKL, mainly expressed in the liver, kidney, and intestine [30,31]. In contrast, the *PKM* gene encodes two isoforms generated through alternative splicing: PKM1 and PKM2. PKM1 is primarily expressed in mature, terminally differentiated tissues such as the heart, brain, and skeletal muscle, with a high demand for ATP production [32]. PKM2, on the other hand, is predominantly expressed in rapidly proliferating cells with high anabolic needs, including embryonic and cancer cells [31,33,34]. The PKM gene spans approximately 32 kilobases (kb) and contains 12 exons and 11 introns. The expression of its two isoforms, PKM1 and PKM2, is regulated by mutually exclusive alternative splicing of exons 9 and 10. The PKM1 transcript is produced by including exon 9 and excluding exon 10, while the PKM2 transcript results from including exon 10 and skipping exon 9 [35].

Among the pyruvate kinase isoforms, pyruvate kinase muscle isozyme M2 (PKM2) functions as a key enzyme in glycolysis [36]. It plays a pivotal role in regulating cellular metabolism, particularly in rapidly proliferating cells such as tumor cells. PKM2 supports tumor progression not only through its metabolic role but also via non-metabolic functions [37,38]. Published studies have shown that PKM2 modulates the glycolytic pathway through its enzymatic activity and transcriptional regulatory functions. This dual functionality enables PKM2 to catalyze the conversion of phosphoenolpyruvate (PEP) to pyruvate in the cytoplasm while also translocating to the nucleus to influence the expression of genes involved in cell proliferation, survival, and metabolic reprogramming. PKM2 influences the glycolytic pathway and broader cellular functions by modulating the activity of several transcription factors, including HIF-1α, signal transducer and activator of transcription 3 (STAT3), octamer binding transcription factor 4 (Oct4), and β-catenin [39,40,41]. PKM2 contributes to the transcriptional activation of genes involved in metabolism, proliferation, and stemness through these interactions [42,43]. Moreover, PKM2 exhibits histone-modifying capabilities like promoting histone H3 phosphorylation, which enhances the expression of oncogenic targets such as cyclin D1 and cellular Myc (c-Myc) [44]. These regulatory functions collectively contribute to tumor cell proliferation, cell-cycle progression, and tumorigenesis. Several recent studies have identified PKM2 as a promising therapeutic target for various diseases, including cancer [45,46], metabolic disorders [47], neurodegeneration [48], neuropathic pain [49], arthritis [50], inflammatory conditions [51] and CVDs, including MI [52,53,54], cardiac hypertrophy [55], and right ventricle failure [56], etc. Therefore, the multifaceted features of PKM2 make it an interesting and unique target for cardiac repair.

## 3. Role of PKM2 in Non-Cardiac Repair

Several recently published studies have highlighted the critical role of pyruvate kinase M2 (PKM2) in non-cardiac repair [57,58,59]. The expression of PKM2 is significantly upregulated during the inflammatory phase of wound healing, while PKM1 remains unchanged. Immunohistochemistry and RNA in situ hybridization analyses of the study have shown that PKM2 expression is predominantly localized in keratinocytes within the hyperproliferative part of the epithelium. Notably, upregulated PKM2 expression in wound keratinocytes was closely linked to angiogenesis during skin repair, both in vivo and in vitro [57]. A study by Liu et al. also demonstrated that the involvement of PKM2 in wound healing by regulating collagen XVII (COL17) expression. Mechanistically, PKM2 promotes STAT3 phosphorylation, enabling co-translocation of PKM2 and STAT3 into the nucleus, where they activate transcriptional programs that enhance keratinocyte function. Additionally, treatment of a PKM2 activator, succinyl-5-aminoimidazole-4-carboxamide ribose (SAICAR), promotes nuclear PKM2, which further increases COL17 expression, leading to improved wound healing outcomes in diabetic *db/db* mice [58]. These findings suggest that targeting PKM2 signaling may offer therapeutic potential for enhancing wound repair under diabetic conditions. Furthermore, combined treatment with KY19382, a Wnt/β-catenin signaling activator, and TEPP-46, an allosteric activator of PKM2, has been shown to significantly accelerate wound healing. This is accompanied by increased expression of markers related to cell proliferation, epithelial regeneration, myofibroblast activation, and angiogenesis within the wound bed [59]. In addition to its intracellular roles, extracellular PKM2 secreted by infiltrating and activated neutrophils has also been shown to facilitate early wound healing by promoting angiogenesis at the injury site [60]. Beyond cutaneous repair, a study by Ryu et al. reported that PKM2 enhances hair regrowth and wound-induced hair follicle neogenesis through activation of the Wnt/β-catenin signaling pathway [61]. Moreover, PKM2 was also found to support skeletal muscle regeneration by promoting the proliferation of skeletal muscle progenitor cells (MPCs) [62].

Apart from this, the involvement of PKM2 in liver regeneration has also been documented in the literature. A study by Chatzipanagiotou et al. (1985) reported that PKM2 is predominantly localized in the periportal zone of the liver after partial hepatectomy [63]. More recent findings indicate that nuclear accumulation of PKM2 plays a critical role in activating STAT3, a key transcription factor required for hepatocyte proliferation and liver regeneration. Inhibition of nuclear PKM2 accumulation using small molecule ML-265 significantly suppressed cell proliferation and exacerbated liver damage. These results suggest that the pro-regenerative effects of PKM2 in the liver are mediated through STAT3-dependent transcriptional regulation [64]. These indicated that the modulation of PKM2-STAT3 signaling could be a therapeutic strategy to enhance liver regeneration. Moreover, upregulated PKM2 was also observed in adult neural stem/progenitor cells. Knockdown of PKM2 led to reduced adult hippocampal neurogenesis and impaired cognitive functions [65]. However, treatment of recombinant PKM2 (rPKM2) reduced ischemic stroke by enhancing angiogenesis, neurogenesis, and functional recovery [66].

**Figure 2:**
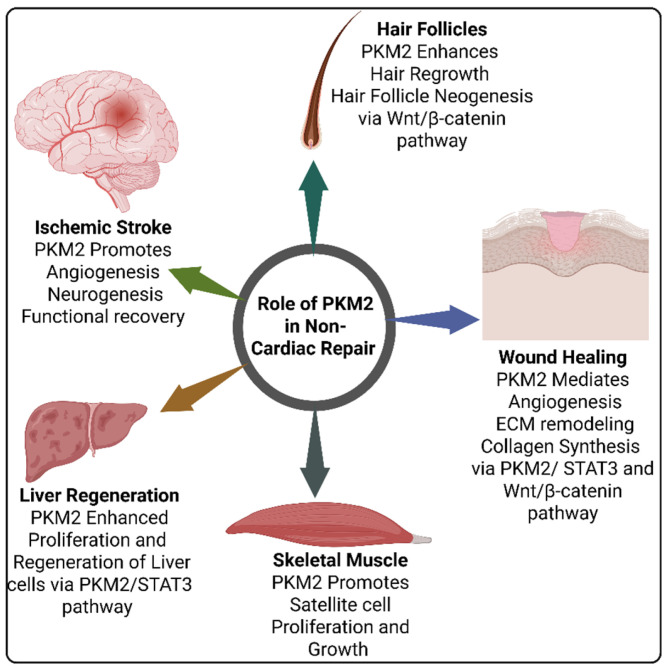
The role of PKM2 in non-cardiac tissue repair. Figure is created with BioRender.com.

In conclusion, PKM2 mediates angiogenesis and collagen XVII production through activation of the STAT3 and Wnt/β-catenin pathways during wound healing. Additionally, PKM2 contributes to hair regrowth and skeletal muscle regeneration by promoting cell proliferation. However, nuclear PKM2 activates STAT3 to drive hepatocyte proliferation and regeneration following injury, suggesting that targeting the PKM2–STAT3 signaling axis could be a promising therapeutic strategy in liver injury. PKM2 also supports neurogenesis and functional recovery in the brain after ischemic stroke, further underscoring its role in tissue repair. Overall, all these findings cover the PKM2 as a multifaceted function in regeneration and highlight its therapeutic potential in various non-cardiac tissue repair contexts. However, as current studies remain limited, further detailed investigation is still necessary to fully elucidate the role of PKM2 in non-cardiac tissue repair and regeneration. The role of PKM2 in non-cardiac tissue repair is illustrated in Figure 2. In the following sections, this review discusses the detailed role of PKM2 in cardiac repair.

## 4. Role of PKM2 in Cardiac Repair and Regeneration

The adult mammalian heart has a limited ability to regenerate, largely because CMs permanently withdraw from the cell cycle shortly after birth [67,68,69]. Recent research efforts have increasingly focused on uncovering and characterizing targets that may enhance the heart’s regenerative response following injury. One of the particularly interesting targets is PKM2, which has shown promising potential in promoting cardiac repair and regeneration. PKM2 is abundantly expressed in the developing heart; however, its expression significantly decreases as the heart matures into an adult [70]. The subsequent sections explore the functional role of PKM2-mediated cardiac repair and regeneration.

## 5. PKM2-Mediated CM Proliferation and Repair

Adult CM lose their proliferative capacity during early postnatal development. In contrast, studies in neonatal mice and pigs (postnatal day 1, P1) have demonstrated that CMs have the capability to regenerate after injury [71]. The regenerative response is largely attributed to the induction of CM proliferation, which is rapidly lost in the adult heart [72,73]. The ability of CMs to proliferate is governed by cell cycle activity and mitotic processes, which are intricately controlled by a network of regulatory genes [74]. Over the past two decades, numerous studies have explored various strategies to induce CM proliferation using proteins, viral vectors, small molecules, and transgenic models [75]. However, these approaches have some limitations such as short or prolonged half-lives, complex modes of administration, a lack of CM specificity, and the potential for adverse effects [76].

In the context of CM proliferation, a study by Magadum et al. (2020), published in Circulation, identified a critical role for PKM2 in promoting CM proliferation and cardiac repair [70]. PKM2 is well known for its high expression in cancer and during embryonic development. A similar pattern of elevated PKM2 expression was observed in the developing heart at embryonic day 16.5 (E16.5), followed by a significant decline as the heart matured into adulthood. Additionally, Magadum et al. employed an inducible, CM-specific PKM2 knockout strategy to delineate the critical role of PKM2 in regulating embryonic CM proliferation and cardiac development. Interestingly, the hearts of E18 knockout mice displayed reduced size, a lower heart-to-body weight ratio, fewer CMs, decreased expression of cell cycle markers, increased levels of the cell cycle inhibitor p27, downregulated PKM2 downstream targets expression including β-catenin, c-Myc, and CCND1, and elevated markers of cardiac hypertrophy such as ANP and BNP [70]. Together, these findings confirm that PKM2 is essential for CM cell cycle, proliferation, and normal heart development.

To restore CM-specific PKM2 expression, Magadum et al. developed a novel circuit-based modified mRNA (modRNA) system enabling CM-specific modRNA (_CMS_modRNA) delivery [70]. This targeted approach led to a selective increase in PKM2 expression within CMs without affecting non-CM cells, including fibroblasts, smooth muscle cells, immune cells, and endothelial cells. Moreover, Magadum et al. further tested the influence of PKM2 on cell cycle marker expression and its ability to promote CM division under both in vitro and in vivo conditions [70]. PKM2 modRNA delivery to isolated CMs from E18 fetal PKM2 knockout mice and neonatal rat CMs resulted in a significant increase in CM numbers and proliferation markers (evidenced by increased percentages of BrdU-positive CMs, as well as very high level of phospho-histone H3 (pH3), Ki67, and Aurora B positive CMs). These findings suggest that CM-specific delivery of PKM2 modRNA delivery has the potential to induce post-neonatal CM proliferation [70].

The findings from the in vitro experiments were further confirmed through a lineage-tracing approach using the Rosa26^^mTmG^ mouse model in combination with _CMS_modRNAs to generate permanently GFP-labeled CMs. Their results revealed that the number of GFP-labeled CMs in mice treated with _CMS_PKM2 modRNA was significantly higher compared to controls in both 3 and 28 days post-MI. These results suggest that overexpression of PKM2 through _CMS_PKM2 modRNA delivery promotes the division of pre-existing CMs, recapitulating the intrinsic regenerative capacity observed in neonatal mice shortly after birth. Notably, at 28 days post-treatment, these CMs exhibited elevated expression of key cell cycle markers, including pH3 and Ki67. Moreover, intramyocardial delivery of _CMS_PKM2 modRNA in the MI model further confirmed the involvement of PKM2 in promoting CM proliferation. Moreover, the findings observed in the Rosa26^^mTmG^ mouse model were further validated using another lineage-tracing system model: the Cre-recombinase-dependent mosaic analysis with double markers (MADM) mouse model. Interestingly, intramyocardial delivery of _CMS_PKM2 modRNAs significantly increased the proportion of the single-color-labeled CMs (more than 8% eGFP^+^ or DsRed^+^ CMs by _CMS_PKM2 modRNA compared to less than 1% by _CMS_Luc modRNA delivery) primarily within the border zone and infarct regions in 14 days of post-MI. Most of these labeled CMs were mononuclear (more than 90%). The results of two independent CM lineage-tracing in vivo models concluded that PKM2 promotes CM cell division [70].

Moreover, a published report demonstrated the role of extracellular PKM2 (EcPKM2) in CM proliferation and cardiac repair. Systemic treatment with the recombinant PKM2 mutant (G415R) protects CMs from apoptosis, promotes CM proliferation, and reduces plasma troponin levels. Interestingly, G415R treatment promotes CM proliferation during the first 1 to 7 days after myocardial infarction (MI), while the percentage of left ventricular scar size and apoptosis is significantly reduced till 30 days post-MI. The authors proposed that G415R mediates its reparative effects through interaction of EcPKM2 with integrin αvβ3, which activates the integrin–FAK–PI3K signaling axis and suppresses phosphatase and tensin homolog (PTEN) expression. Modulation of the EcPKM2–integrin–FAK–PI3K pathway enhances CM proliferation by reducing apoptosis and decreasing cardiac fibrosis [77].

In addition, the involvement of PKM2 in cardiac repair has also been demonstrated in large animal models recently. A study by Wei et al. (2024) showed that recombinant human checkpoint kinase 1 (CHK1) promoted CM survival, proliferation, and myocardial repair in a porcine model of myocardial ischemia/reperfusion (I/R) injury via PKM2 [78]. To confirm the role of PKM2, Wei and colleagues employed PKM2 silencing strategies in a myocardial infarction model, using three experimental groups: I/R+hydrogel, I/R+rhCHK1-hydrogel, and I/R+rhCHK1+PKM2 inhibitor (PKM2i). Notably, the I/R+rhCHK1+PKM2i group exhibited significantly reduced cardiac function, decreased expression of cell cycle markers (Ki67^+^, pH3^+^, and Aurora B^+^) in CMs, and increased fibrosis and apoptosis. Mechanistically, CHK1 was shown to directly bind to PKM2, activating it through phosphorylation at serine 37 (S37) and tyrosine 105 (Y105). The authors proposed that this interaction promotes metabolic reprogramming, thereby contributing to enhanced cardiac repair.

The overall proliferation of CMs is also supported by the interaction of the PPP, which plays a central role in nucleotide and DNA synthesis by generating ribose-5-phosphate (R5P), a critical sugar backbone used in the formation of nucleotides [79,80]. During the non-oxidative phase of the PPP, intermediates of glycolysis—specifically fructose-6-phosphate and glyceraldehyde-3-phosphate—are enzymatically converted into ribose-5-phosphate. This R5P is then used by phosphoribosyl pyrophosphate synthetase (PRPS) to produce phosphoribosyl pyrophosphate (PRPP), a key precursor for the synthesis of purine and pyrimidine nucleotides, which are essential for DNA and RNA synthesis [81]. [U13C] glucose flux using mass spectrometry in P3 neonatal rat CMs transfected with Luc or PKM2 modRNA showed significantly increased levels of nucleosides (AMP, CMP, GMP, and UMP), which support DNA synthesis and CM proliferation [70].

The chronic effects of intramyocardial delivery of _CMS_PKM2 modRNAs were investigated by Magadum et al. [70]. Notably, their study demonstrated that _CMS_PKM2 modRNA treatment significantly improved cardiac function, reduced scar size, increased CM numbers, and attenuated adverse cardiac remodeling after 28 days of post-MI. Furthermore, _CMS_PKM2 modRNA delivery in MI mice enhanced the expression of cell cycle markers and improved overall survival rates. Overall, their study suggested _CMS_PKM2 modRNA delivery promotes CM proliferation in both acute and chronic MI conditions by prolonging CM survival, enhancing angiogenesis, and reducing oxidative stress and apoptosis.

In the study by Nakada et al. (2022), single-nucleus RNA sequencing of neonatal pig hearts following apical resection revealed a transient regenerative window characterized by enhanced CM proliferation and metabolic remodeling [82]. Notably, PKM2 emerged as one of the key metabolic regulators upregulated during this regenerative phase. PKM2, a glycolytic enzyme known for its dual roles in energy metabolism and gene regulation, showed increased expression in proliferating CM within the resected apex. This upregulation is consistent with previous findings that link PKM2-mediated metabolic reprogramming to anabolic support for CM proliferation [70]. The study suggests that the induction of PKM2 may facilitate the metabolic shift from oxidative phosphorylation to aerobic glycolysis, a hallmark of proliferative states, thereby enabling CM cell cycle re-entry. These findings underscore the importance of metabolic plasticity, particularly PKM2-associated glycolytic remodeling as a critical determinant of cardiac regeneration in large mammals and position PKM2 as a potential target for therapeutic strategies aimed at enhancing myocardial repair.

**Figure 3:**
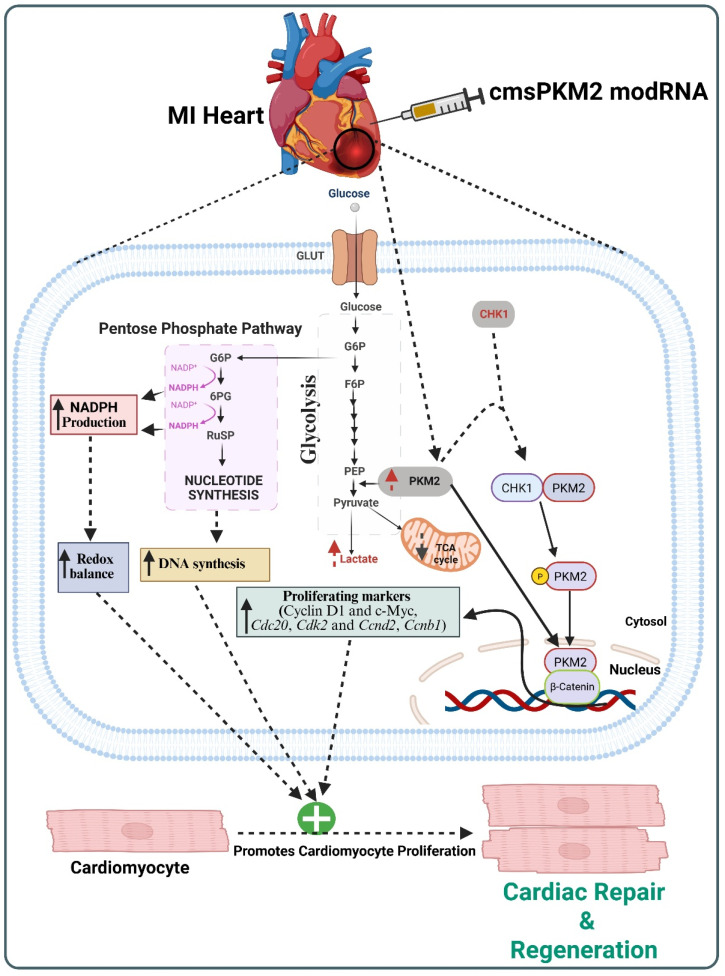
PKM2-induced molecular mechanism in CM proliferation and cardiac repair. Figure is created with BioRender.com.

In conclusion, these major findings suggest that PKM2 plays a crucial role in modulating CM proliferation and cardiac repair, as demonstrated in preclinical mouse and porcine models of MI. The multifaceted role of PKM2 in CM proliferation and cardiac repair is illustrated in Figure 3.

## 6. PKM2 Modulates Oxidative Stress in the Heart

As PKM2 plays a multifaceted role in cellular metabolism, it can regulate total and mitochondrial ROS levels, apoptosis, and key biosynthetic processes [83]. In addition to that, PKM2 also functions as a molecular integrator of metabolic dysfunction, oxidative stress, lipid metabolism, and tissue inflammation [84,85]. Elevated ROS levels have been found to reduce the postnatal proliferative window of CM, whereas inhibition of ROS can extend this window and delay cell cycle arrest [86]. A report by Lee et al. (2024) reported that deletion of PKM2 in CMs resulted in a significant decrease in total ATP levels and an increase in both ROS and mitochondrial superoxide in global PKM2 knockout (PKM2^−/−^) mice [87]. These alterations were associated with dysregulated glucose metabolism and compromised mitochondrial function, ultimately contributing to increased oxidative stress. Moreover, PKM2 is known to facilitate anabolic coupling with the PPP, supporting proliferative cell growth and modulating ROS levels and DNA damage [88,89,90]. In line with this, Magadum et al. (2020) demonstrated that overexpression of PKM2 in CMs redirects glycolytic flux toward the PPP anabolic route that supports cell proliferation [70]. In a mouse model of MI, intracardiac delivery of _CMS_PKM2 modRNA resulted in upregulation of glucose-6-phosphate dehydrogenase (G6PD), the rate-limiting enzyme of the PPP, leading to enhanced nicotinamide adenine dinucleotide phosphate (NADPH) production, reduced ROS accumulation, and diminished DNA damage as early as two days post-MI. NADPH generated through the PPP plays a key role in maintaining mitochondrial function and preventing ROS-induced apoptosis by regenerating reduced glutathione (GSH) [91]. High-performance liquid chromatography (HPLC) analysis of infarcted hearts treated with _CMS_PKM2 modRNA revealed a significantly elevated GSH/GSSG ratio and lower ROS levels compared to _CMS_Luc modRNA injected mice heart. Furthermore, assessment of DNA damage markers showed a marked decrease in 8-hydroxyguanine (8-OHG) and phosphorylated ATM (pATM) levels, indicating reduced oxidative DNA damage and a suppressed DNA damage response, respectively [70]. Collectively, these findings suggest that _CMS_PKM2 modRNA delivery promotes cardiac repair by enhancing PPP activity, thereby supporting CM proliferation while mitigating oxidative stress and DNA damage. In proliferating cells, such as regenerating CMs, increased PPP activity ensures a sufficient supply of both nucleotides for replication and NADPH for maintaining redox balance, thereby coupling anabolic biosynthesis with antioxidant defense.

## 7. PKM2 Role in Cardiac Fibrosis and Hypertrophy

Recently, PKM2 has been identified as a novel biomarker for heart failure [92]. Satyanarayana et al. demonstrated that PKM2 promotes collagen synthesis and secretion in myofibroblasts by redirecting glycolytic intermediates toward de novo glycine synthesis [93]. Pharmacological modulation of PKM2 via small molecules conferred cardioprotection by attenuating fibrosis in pathological hypertrophy models like isoproterenol (ISO)-induced cardiac hypertrophy and angiotensin-II mediated cardiac remodeling [94,95]. The CM-specific deletion of *PKM2* significantly impairs cardiac function, while *PKM2* overexpression attenuates transverse aortic constriction (TAC)-induced cardiac hypertrophy and enhances overall cardiac performance [55]. Moreover, overexpression of PKM2 via intramyocardial injection of _CMS_PKM2 modRNA showed significant improvement in cardiac function and cardiac remodeling in post-MI mice. Magnetic resonance imaging and echocardiography experiments revealed that _CMS_PKM2 significantly increased the ejection fraction percentage (more than 30% increase in %EF _CMS_PKM2 modRNA injected mice compared to _CMS_Luc) and the percentage fractional shortening after 28 days of MI. Furthermore, _CMS_PKM2 modRNA also resulted in higher heart weight to body weight ratio as a result of new muscle formation, reduction in fibrotic tissue (by more than 50%) compared with _CMS_Luc modRNA injected mice [70]. Notably, similar results were also reported with extracellular PKM2 (EcPKM2) in MI and IR animal models. Treatment with the PKM2 mutant (G415R) resulted in a significant reduction in infarct scar sizes, cardiac remodeling, and preserved myocardial structure in both MI and IR mice [77]. Interestingly, the findings from the mouse model were also confirmed in a large animal model of IR. A study by Wei et al. (2024) demonstrated that treatment with rhCHK1 in pigs significantly reduced scar size, decreased the expression of fibrosis markers, and improved cardiac function through PKM2 [78]. In conclusion, overexpression of PKM2 through _CMS_PKM2 modRNA and extracellular PKM2 both show remarkable anti-fibrotic roles in MI and IR models. PKM2 also enhances cardiac function, improves remodeling, decreases infarct scar thickness, and maintains myocardial structure in post-MI. These findings are also supported in large animals, all of which highlight the therapeutic potential of PKM2 modulation for promoting cardiac fibrosis, hypertrophy, and functional recovery after MI.

## 8. Molecular Mechanisms of PKM2 in Cardiac Repair

PKM2 is a key glycolytic enzyme that plays crucial roles beyond its canonical metabolic function. In the nucleus, PKM2 functions as a protein kinase, interacting with transcription factors such as HIF-1α, β-catenin, STAT3, and Oct4, which in turn influence the expression of genes involved in cell proliferation, angiogenesis, and survival [39,40,41]. Moreover, recent studies showed that PKM2 also plays a crucial role in cardiac repair by regulating various pathways, including cellular metabolism, gene expression, and signaling mechanisms, which all support the CM survival, proliferation, and regeneration after myocardial injury. The PKM2 regulates CM metabolism, shifting from oxidative phosphorylation to aerobic glycolysis after injury, a phenomenon known as the Warburg effect. This metabolic reprogramming mediated by PKM2 benefits CMs in different ways, i.e., increased ATP production and a redirection of glycolysis towards the PPP. The coupling of glycolysis with PPP further supports proliferating CMs by increasing production of NADPH, protecting proliferating CMs from oxidative stress by increasing glutathione, and reducing ROS-induced apoptosis. Moreover, PKM2 also acts as a protein kinase after translocating to the nucleus and interacts with transcription factors, with transcriptional factors such as β-catenin, leading to the activation of cell proliferation-associated genes c-Myc and Cyclin D1. Moreover, checkpoint kinase 1 (CHK1) also directly interacts with PKM2 and phosphorylates it at serine 37 (S37) and tyrosine 105 (Y105). This phosphorylation facilitates downstream metabolic reprogramming and induces nuclear translocation of the phosphorylated PKM2 (pPKM2), thereby promoting metabolic reprogramming, CMs proliferation, and myocardial repair. In addition, a published report demonstrated that PKM2 interacts with hypoxia-inducible factor 1-alpha (HIF-1α), facilitating the isoform shift from PKM1 to PKM2 under myocardial infarction (MI) conditions [96]. This isoform transition may play a critical role in supporting the metabolic and proliferative needs of CMs under hypoxic stress. Furthermore, PKM2 was also found to be colocalized with angiogenesis marker vascular endothelial growth factor (VEGF) in wound keratinocytes [57]. This suggested potential role of the PKM2/HIF-1α/VEGF axis in the angiogenesis process. However, the PKM2/HIF-1α/VEGF axis in the context of cardiac repair needs further investigation.

### 8.1. Interaction of PKM2 with Beta-Catenin (β-Catenin)

β-catenin is a multifunctional protein that plays essential roles in both cell–cell adhesion and gene transcription, especially within the Wnt signaling pathway. Additionally, β-catenin serves as a key nuclear effector in canonical Wnt signaling by regulating the expression of target genes. In cancer, nuclear PKM2 functions as a protein kinase and forms a complex with β-catenin, which transactivates genes involved in cell cycle regulation. Similarly, in the context of cardiac repair, a study by Magadum et al. (2020) demonstrated that PKM2 directly interacts with β-catenin, which was confirmed by coimmunoprecipitation assay in neonatal rat CMs [70]. Moreover, luciferase assay showed that PKM2 and β-catenin are co-localized in CMs nuclei. Direct interaction of PKM2 and β-catenin leads to the upregulation of β-catenin target genes such as *cyclin D1* and *c-Myc*. This transcriptional activation promotes CM proliferation and prevents cell cycle arrest. Furthermore, elevated *c-Myc* expression enhances PKM2 levels, suggesting a positive feedback loop that sustains CM proliferation. This suggests that the interaction of PKM2 and β-catenin plays a significant role in CM proliferation and cardiac repair.

### 8.2. Interaction of PKM2 with Checkpoint Kinase 1 (CHK1)

Checkpoint kinase 1 (CHK1) is a serine/threonine-specific protein kinase that plays a pivotal role in maintaining genomic stability by regulating the cell cycle and the DNA damage response (DDR). CHK1 also acts as a signal transducer which activates cell cycle checkpoints, particularly the G2/M checkpoint. In addition, CHK1 is involved in DNA repair mechanisms and cell apoptosis. Wei et al. (2024) demonstrated that administration of recombinant human CHK1 (rhCHK1) protein encapsulated in hydrogel significantly stimulated CMs proliferation and attenuated cardiac inflammatory responses at both 3 and 28 days following I/R injury [78]. Mechanistically, CHK1 interacts directly with PKM2 in the cytoplasm, promoting its phosphorylation at serine 37 (S37) and tyrosine 105 (Y105). These phosphorylation events facilitate nuclear translocation of PKM2, which subsequently activates the transcription of cell cycle-related genes and supports metabolic reprogramming, ultimately promoting cardiac repair and regeneration. These findings highlight the CHK1–PKM2 axis as a novel and critical pathway in the regulation of CM proliferation and cardiac regeneration following injury.

### 8.3. Interaction of PKM2 with Integrin α_v_β3

Integrin alpha-v beta sub-3 (α_v_β3) is a heterodimeric transmembrane glycoprotein also known as vitronectin receptor, which plays a critical role in cell adhesion and signal transduction [97]. Huang et al. demonstrated that systemic administration of a recombinant PKM2 mutant (G415R) protects CMs from apoptosis, promotes CM proliferation, reduces cardiac fibrosis, and improves cardiac function. Mechanistically, co-immunoprecipitation experiments revealed that extracellular PKM2 (EcPKM2) interacts with integrin αvβ3 on CMs and activates the integrin–FAK–PI3K signaling axis, which subsequently suppresses phosphatase and tensin homolog (PTEN) expression. This pathway enhances *CM* proliferation by decreasing apoptosis, promoting proliferation, and reducing cardiac fibrosis [77].

### 8.4. Interaction of PKM2 with HIF-1α

PKM2 and HIF-1α are involved in a positive feedback loop under hypoxic conditions: HIF-1α stabilizes and activates PKM2 transcription by binding to hypoxia response elements in its promoter. In turn, PKM2 translocates to the nucleus and coactivates HIF-1α, thus amplifying the hypoxic response [40,98,99]. Elevated HIF-1α has been shown to upregulate angiogenic factors like VEGFA under ischemic conditions [100]. c-Myc, a direct PKM2 target, drives CM cell cycle activity [70,101] and is required for proper coronary vascular formation [102]. NF-κB activation contributes to angiogenic cytokine expression and vascular growth [103]. Together, these factors can explain the proliferation, vascular remodeling, and cardiac protection provided by hPKM2 treatment. PKM2 functions as a transcriptional co-activator of HIF-1α and in ischemic CMs, this interaction has been shown to support metabolic reprogramming, reduce apoptosis, and enhance cell survival, indicating its CM-specific relevance beyond tumor biology.

## 9. Therapeutic Application of PKM2 in Cardiac Repair

Cardiovascular diseases (CVDs) represent a significant global health burden, accounting for a substantial proportion of morbidity and mortality worldwide [104]. Although several therapeutic options, including beta-blockers, diuretics, calcium channel blockers, and thrombolytic agents such as streptokinase, are already available to treat CVDs, these treatments have notable limitations [105,106,107,108]. So, there is an urgent need to develop novel therapeutic strategies to address this unmet need. Gene therapies mainly work through introducing genetic material into cells or tissues. These genetic changes may either replace dysfunctional variations with functioning ones, provide new functions, or improve already-existing ones [109]. After the development of COVID-19 mRNA-based vaccines, mRNA therapy has emerged as a promising tool to develop therapeutics [110,111,112]. Moreover, mRNA-based therapies offer several advantages, including low immunogenicity, ease of dose adjustment, and no risk of permanent genomic alteration. These benefits have garnered significant attention, positioning nucleic acid-based products as a promising therapeutic approach in recent years [76,112,113,114,115,116,117]. The application of Specific Modified mRNA Translation System (SMRTs) for PKM2 mRNA delivery represents a promising strategy to achieve CM-specific protein expression. SMRTs exploit the unique microRNA expression profile of cell or cardiomyocyte by incorporating complementary target site microRNA into the mRNA construct [118]. This design ensures that PKM2 translation is actively suppressed in off-target cells such as fibroblasts, or immune cells while allowing robust expression specifically in CMs (more than 98% of transfected cells are CMs) in the heart. Such precision significantly enhances the spatial control of therapeutic mRNA activity, reducing potential side effects and improving the safety and efficacy of PKM2-based regenerative interventions [118]. Importantly, SMRTs also enable scalable and reproducible manufacturing with more predictable dose–response relationships, which will be critical for clinical translation. Regarding delivery strategies, intramyocardial injection offers high local expression but is invasive and best suited for proof-of-concept or surgical settings, whereas intracoronary infusion provides broader myocardial distribution and is more clinically translatable for large animals and early-phase human studies, though it may be limited by lower transfection efficiency and greater off-target exposure. Balancing these delivery modalities, alongside precise dose control through SMRTs engineering, will be essential to maximize efficacy while minimizing risks in large animal models and future clinical trials.

As PKM2 is an emerging therapeutic potential target for CM proliferation and cardiac repair due to its dual functionality, i.e., metabolic regulation and gene transcription. In the injured heart, PKM2 promotes CM proliferation, reduces oxidative stress, and supports cardiac tissue regeneration by shifting glycolytic flux toward the PPP, thereby enhancing NADPH production and reducing ROS. A recent study showed that CM-specific overexpression of PKM2 by modRNA delivery systems resulted in enhanced CM proliferation, improved cardiac function, and reduced scar size post-MI and heart failure (HF). Moreover, PKM2 also showed interaction with key regulatory proteins like beta-catenin, CHK1, and integrin α_v_β3. Further amplifying its reparative potential by modulating cell cycle pathways, ROS, and DNA damage pathways. These findings suggest that therapeutic modulation of PKM2, particularly via gene or protein delivery systems, holds significant potential for treating ischemic heart disease and promoting myocardial regeneration. However, further clinical studies are still necessary to fully validate its safety, efficacy, and long-term outcomes (Table 1).

## 10. Challenges and Knowledge Gaps

PKM2 has emerged as a promising therapeutic target for cardiac repair due to its multifaceted roles in metabolic regulation, transcriptional control, and tissue regeneration. Studies have demonstrated that both intracellular overexpression and extracellular forms of PKM2 can enhance cardiac function, reduce infarct scar size, and preserve myocardial structure in various small animal models of MI, HF and IR injury, as well as in large animal models of CVD. Despite these encouraging findings, significant challenges remain. For example, Magadum et al. demonstrated that intracardiac delivery of _CMS_PKM2 modRNA improves outcomes in mice. While intracardiac delivery is clinically feasible (e.g., during cardiac surgery or catheter-based interventions), there is a need to develop safer and more efficient delivery strategies for large animals and humans to ensure targeted myocardial expression without off-target effects, systemic toxicity, or inconsistent responses. Approaches such as SMRTs offer precise CM-specific expression, minimizing off-target translation. Repeated _CMS_PKM2 modRNA delivery may further improve outcomes; however, the risks of immunogenicity or cumulative toxicity must be carefully evaluated. Moreover, although preclinical studies suggest PKM2 promotes CM proliferation without inducing arrhythmias or hypertrophy, these potential risks should be systematically addressed in future studies. Importantly, oncogenic risks associated with PKM2′s nuclear functions must also be rigorously investigated before clinical translation. Ultimately, further research is needed to optimize dosing, refine targeted delivery methods, and conduct clinical studies to validate the therapeutic safety and efficacy of PKM2 in cardiac regeneration.

## 11. Conclusions and Future Directions

PKM2 plays a multifaceted role in cardiac regeneration and repair by regulating cellular metabolism, redox status, and gene transcription. PKM2 can promote CM proliferation, suppress oxidative stress via activation and coupling with the PPP, and interact with key master regulator signaling proteins such as β-catenin, CHK1, HIF-1α, and integrin α_v_β3. Cardiac-specific overexpression of PKM2 via modRNA delivery has been found to result in significant enhancement of cardiac function, reduction in DNA damage, and enhancement of myocardial regeneration in mouse models of MI and HF. Moreover, RNA-based therapies offer several advantages, such as low immunogenicity, ease of dose adjustment, and no risk of permanent genomic alteration. In addition to this, modRNA therapy platforms like SMRTs further minimized translational challenges of targeting PKM2, including issues related to specificity, off-target effects, metabolic rewiring in other organ for RNA based therapy. Collectively, these findings position PKM2 as a central regulator of metabolic and regulatory pathways in cardiac repair. However, further translational research is justified to establish its complete therapeutic potential for clinical application in the management of heart injury.

## Figures and Tables

**Table 1:** Therapeutic approaches/modalities used for PKM2 activation or delivery to the heart.

S. No	Therapeutic Modalities	Disease Model	Delivery	Effect	Advantages	Limitations	Reference
1.	PKM2 modRNA	MI and HF in Mice	Intramyocardial injections of PKM2 modRNA	CM-specific PKM2 delivery induced CM proliferation, angiogenesis, reduced CM death, improved cardiac function and mice survival	Transient, highly specific and effective, dose-controlled, safe, no genomic integration, and reduced immune response	No long-term expression	[70]
2.	Recombinant human checkpoint kinase 1 (CHK1)	MI in Porcine Model	Intramyocardial injections of non-loaded rhCHK1--hydrogel	CHK1-mediated metabolic reprogramming promotes CM proliferation and cardiac repair by binding with PKM2	Direct and local delivery of recombinant rhCHK1—hydrogel	Very short-term and non-cell specific expression.	[78]
3.	Recombinant PKM2 mutant (G415R)	MI/IR injury	Intraperitoneal (IP) injection	Treatment of G415R preserves CMs and reduces cardiac fibrosis in post-MI injury	Systemic, multi-dose delivery	Very short-term and non-specific expression	[77]

## Data Availability

No new data were created or analyzed in this study.
